# Efficacious elimination of crystal violet pollutant via photo-Fenton process based on Gd_(2−x)_La_(x)_Zr_2_O_7_ nanoparticles

**DOI:** 10.1038/s41598-023-34838-w

**Published:** 2023-05-12

**Authors:** M. Abdelbaky, A. M. Abdelghany, A. H. Oraby, E. M. Abdelrazek, M. M. Rashad

**Affiliations:** 1 Faculty of Science, New Mansoura University, International Coastal Rd, New Mansoura City, Dakahlia Egypt; 2 Spectroscopy Department, Physics Research Institute, National Research Centre, 33 ElBehouth St., DokkiGiza, 12311 Egypt; 3 Physics Department, Faculty of Science, Mansoura University, Mansoura, 35516 Egypt; 4 Electronic and Magnetic Materials Department, Advanced Materials Institute, Central Metallurgical Research and Development Institute (CMRDI), P.O. Box 87, Helwan, 11421 Cairo Egypt

**Keywords:** Biophysics, Materials science, Nanoscience and technology, Physics

## Abstract

The photo-Fenton process is an appropriate method of the Advanced Oxidation Process that is used in the photocatalysis of organic dyes like crystal violet (CV). La^3+^ ion substituted gadolinium zirconium oxide Gd_(2−*x*)_La_(*x*)_Zr_2_O_7_ nanopowders (x = 0.1, 0.2, 0.3, and 0.5) have been successfully prepared by using sol–gel auto-combustion method to be used for the efficient photocatalysis of CV with photo-Fenton process. The well-crystallized defect-fluorite, structured with space group: Fm-3m, was detected using X-ray diffraction analysis. The lattice parameters were found to increase with the evaluated La^3+^ ion concentration. The grain size of the synthesized powders increased with the increase in La^3+^ ion content. The SAED patterns depicted fluorite structured fluorite. UV/Vis. spectrophotometer was used for the determination of band gap energy of Gd_(2−*x*)_La_(*x*)_Zr_2_O_7_ nanopowders which increased with increasing La^3+^ ion content. It was found to enhance from 4 to 3.6 eV. The visible spectrophotometer was used for determining unknown concentrations during the photocatalysis process to assure the effectiveness of the process. Overall, results illustrate that the photo-Fenton reaction on Gd_(2−x)_La_(x)_Zr_2_O_7_ performed excellently in removing crystal violet (CV). The photo-remediation ratio of CV reached 90% within 1 h.

## Introduction

The paper, textile, leather, plastic, electroplating, food processing, pharmaceutical, and agricultural sectors are just a few of the industries that have seen a rise in commerce in the contemporary period^1,2^. Because these sectors produce high-quality products, they are significant to society as a whole. The majority of industrial operations rely on organic dyes to color their goods, and then they discharge these colors into freshwater aquifers, such as streams and rivers, which eventually empty into the sea^3–5^.

These dyes have been there for a long time, and not only are they harming nearly every type of life seriously, but they are also upsetting the natural equilibrium^6,7^. About 20% of these colors are dumped into the environment as effluents, harming the ecology. These pollutants are exceedingly difficult to decompose due to their stable structure^8,9^. Additionally, these pollutants can lead to major issues in people, such as eye irritation, skin allergies, genetic mutations, and liver issues^10–12^.

One of these hazardous dyes, known as crystal violet (CV), is used in a variety of goods made by Gram, including fertilizers, detergents, bacteriostatic agents, colors for leather, and detergents. It is a potent carcinogen for marine creatures. Additionally, CV destroys chromosomes, which results in serious problems when the injured cells divide. Furthermore, CV dye is classified as triphenylmethane cationic dye which is utilized in textile and paper dye sectors. Various products, including fertilizers, antifreeze, detergents, and leather, are also colored using this technique. As a histology stain, CV is also used, most notably in Gram staining to classify the bacteria^13,14^.

Aquatic life disturbance and water contamination are caused by dye dumping in wastewater^15^. As a result, a suitable and efficient method for treating wastewater containing dyes, such as CV^16^, is required due to the dye's proven ability to cause cancer and other mutations in both people and animals^17^ and humans is essential. Traditional oxidants and coagulants, as well as biodegradation, coagulation, adsorption, and physical deposition, proved insufficient for treating CV^18,19^.

On the other hand, better oxidants, microwave catalysis, photocatalysis, membrane technology, and advanced oxidation processes (AOPs) seem promising for CV decolorization^20–22^. The fundamental flaw of physical treatment is that it merely moves dyes from a liquid to a solid form, which is difficult to clean. As a result, for degrading such pollutants, chemical treatment employing AOPs, particularly heterogeneous photocatalysis, has garnered interest ^23^.

According to Wang et al.^24^, metal oxides in heterogeneous AOPs produce powerful, non-selective hydroxyl radicals (HO^•^) that break down a range of organic contaminants into short-chain aliphatic acids, which can be more readily eliminated^25^. In UV–visible light, the electron–hole pair process is necessary to introduce intermediate organic molecules that may completely mineralize at the surface of metal oxide, producing green end products. Since visible light is less expensive than UV light from an economic perspective, it has been extensively studied how new nano-sized photocatalysts respond to it.

In previous works, lots of materials were introduced in the photocatalysis process of dyes contained in wastewater. Nanosphere of TiO_2_, Mn-doped and PVP-capped ZnO NPs, Ag-modified Ti-doped-Bi_2_O_3_, ZnS NPS, CeO_2_-TiO_2_ nanocomposite, AgBr-ZnO nanocomposite, and grafted sodium alginate/ZnO/graphene oxide have all been utilized to decolorize CV^26^. Liu et al.^27^ used PM-Fe/Ni/H_2_O_2_ in the degradation of crystal violet with a Fenton like reaction by Fenton-like reaction. Oladipo et al.^28^ prepared Octadecylamine capped cadmium nanoparticles CdS to be used in the CV photodegradation process. ZnO/CNA was also prepared for the degradation of CV by Messaoudi et al.^29^ and lots of materials are used for the degradation of CV as also other organic dyes.

The performance of the aforementioned oxides will next be evaluated, and the results of the present investigation will be contrasted. In this work, the incorporation of lanthanum with Gd_2_Zr_2_O_7_ was discussed to enhance the value of the energy gap value to be appropriate for the photocatalysis process, especially by the Fenton-like method. The reason why we choose pyrochlore is its good ability in the photodegradation of organic dyes due to its chemical composition that distinguishes it from other compounds. This feature qualifies it to be a good photocatalyst. Since many types of pyrochlore were used before in the analysis processes, we used pyrochlore with new elements Gd_(2−*x*)_La_(*x*)_Zr_2_O_7_ that have distinctive features making them compatible with the purpose of photocatalysis. Gd_(2−*x*)_La_(*x*)_Zr_2_O_7_ are also distinguished from others by more characteristics and that assures the novelty of this work.

To understand deeply the fascinating features of pyrochlore, we must know about its classification and composition. Pyrochlore-structured oxides offer a wide range of compositions that lead to outstanding characteristics and applications in superconductivity, thermal barrier coatings, luminescence, and ferromagnetism. Pyrochlore has the general formula A_2_B_2_O_6_O (A = Y or rare earth; B = Ti, Zr, Hf, Sn, Tc, or Pb)^30^. The pyrochlore structure is a superstructure variant of the simple fluorite structure (AO_2_ = A_4_O_8_, with the A and B cations arranged along the 110 direction). The extra anion vacancy is found in the tetrahedral interstice between two B-site cations. Geometrical frustration and new magnetic effects are particularly vulnerable in these systems. Electronic insulators (e.g., La_2_Zr_2_O_7_), ionic conductors (e.g., Gd_1.9_Ca_0.1_Ti_2_O_6.9_), mixed ionic and electronic conductors, spin ice systems (Dy_2_Ti_2_O_7_), spin glass systems (Y_2_Mo_2_O_7_), and Haldane chain systems (Tl_2_Ru_2_O_7_) are among the physical properties of the pyrochlore structure (Cd_2_Re_2_O_7_). More disordered structures, such as bismuth pyrochlores, have also been investigated due to their fascinating high-frequency dielectric properties.

Interestingly, herein, rare-earth zirconates with the formula Ln_2_Zr_2_O_7_ (Ln = La–Gd) correspond to the pyrochlore structural type, which in turn belongs to the fluorite homologous series. The pyrochlore structure, as it is known, is a variant of the fluorite structure in which 1/8 of the oxygen is removed and oxygen vacancies are somewhat ordered^31–33^. Consequently, the effect of La^3+^-substituted Gd_2_Zr_2_O_7_ on the structural parameters, crystallite size, microstructure, and optical properties was considered. Meanwhile, CV was selected as the target elicited organic contaminant model to test the photo-Fenton performance of La^3+^ -substituted Gd_2_Zr_2_O_7_ nanoparticles.

## Materials and methods

### Preparation of the Gd_(2−x)_La_(x)_Zr_2_O_7_ nanoparticles

Materials used in the preparation process include gadolinium nitrate hexahydrate Gd(NO_3_)_3_. 6H_2_O that was supplied from Alfa Aesar Co., as well as zirconyl oxychloride octahydrate ZrOCl_2_.8H_2_O from Alpha Chemika Co. and tartaric acid COOH(CHOH)_2_COOH from Lanxess Co.

The sol–gel auto-combustion technique will be used to create Gd_(2−x)_La_(x)_Zr_2_O_7_ nanoparticles, where x equals (0.1, 02, 0.3, and 0.5). After mixing, ZrOCl_2_.8H_2_O and GdH_12_N_3_O_15_ were dissolved in 40 ml of distilled water in a 1:1 molar ratio. Tartaric acid, which functions as a fuel, will be added to the mixture on a heater set to 350 °C until sol–gel forms. Following that. Sol–gel will be dried in an oven at 100 °C for 24 h. Finally, the dried sample will be fired in a furnace at 1100 °C for two hours to generate Gd_(2−x)_La_(x)_Zr_2_O_7_ nanoparticles.

### Sample characterization

The phase composition, crystallinity, crystallite size, and structural parameters of the different samples with Cu *K*_α_ radiation were established based on the X-ray diffractometer diffraction patterns (Philips PW 1390). Thermo Scientific's Nicolet iS 10 spectrophotometer is frequently used to measure the Fourier transform infrared (FTIR) absorption spectra of prepared samples at room temperature, which range from 4000 to 400 cm^−1^. The tested sample was created using the compression procedure, which entailed mixing 200 mg of KBr and 2 mg of powdered Gd_(2−x)_La_(x)_Zr_2_O_7_ samples to create a transparent disc. UV/Vis/Near IR spectrophotometry (a Jasco V770, Japan) technique is the technology that is used to investigate the optical properties and optical energy gap. The microstructure of Gd_(2−x)_La_(x)_Zr_2_O_7_ particles was inspected using transmission electron microscopy (Jeol JEM-1011, Japan).

### Photoreactor and photocatalytic measurement

In the visible part of the electromagnetic spectrum, crystal violet has a large absorption band (591 nm). Despite its usage in the textile, culinary, and cosmetic sectors, CV dye can damage aquatic environments.

**Figure 1:**
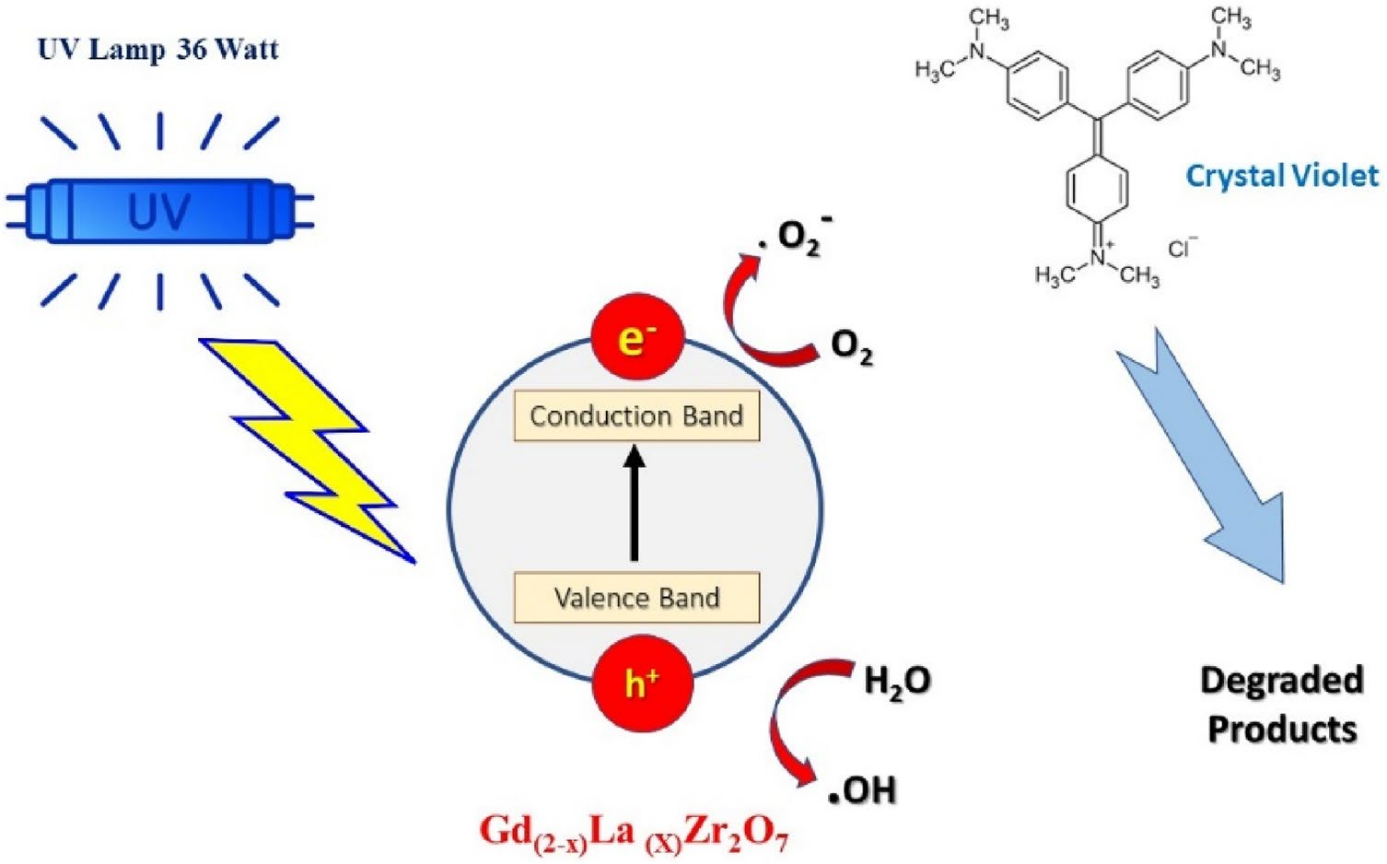
Graphical representation of photocatalysis process of CV using Gd_(2−x)_La_(x)_Zr_2_O_7_ nanoparticles.

As shown in Fig. 1., using Gd_(2−x)_La_(x)_Zr_2_O_7_ nanoparticles as a model, it was chosen as the target organic pollutant to study how it degraded in the presence of UV radiation. The photocatalytic oxidation experiments were carried out in a 100 ml beaker mounted on a magnetic stirrer and exposed to two parallel, 36-Watt UV lamps above the beaker, which was housed in a lamina with silvered inside walls. To ensure uniform photocatalyst dispersion and facilitate the establishment of an adsorption–desorption equilibrium, the catalyst was introduced to a beaker containing 50 mL of an aqueous solution of CV (10 ppm). A solution of 33% H_2_O_2_ in 1 mL was added to the reaction container. The reaction was thought to have started with the addition of H_2_O_2_. At regular intervals during the procedure, liquid aliquots were taken out of the vessel (15 min). Centrifuging was done on the liquid before analysis. The liquid solutions were analyzed using a visible spectrophotometer after the reactions. Based on the organic dye's rate of degradation, kinetic investigations were carried out. The degradation processes of the dye molecules might be expressed as follows using the Langmuir–Hinshelwood model, which adopts that dye degradation follows pseudo-first-order kinetics:

1 $$- {\text{dC}}/{\text{dt}} = {\text{K}}_{{{\text{app}}}} \left[ {\text{C}} \right]$$

Integrating this equation yields the following relationship, with the restriction that C = C_o_ at t = 0, where C_o_ is the initial concentration in the bulk solution after dark adsorption and t is the reaction time.

2 $$- {\text{ln}}\left[ {{\text{A}}/{\text{A}}_{{\text{o}}} } \right] = {\text{K}}_{{{\text{app}}}} {\text{t}}$$

In this equation, Ao stands for the dye's initial absorbance, and T stands for the dye's absorbance at time t. t is the amount of time the dye was exposed to light, and Kapp is the apparent reaction rate constant. The slope of the linear plots on a graph of − ln[At/A_0_] vs. time should be equal to the apparent-first-order rate constant (Kapp). The Kapp values show how quickly photocatalyst materials break down CV molecules when H_2_O_2_ and visible light are present. The CV removal efficiency (η) was determined in the following manner:

3 $$\eta \left( \% \right) = {1}00\left[ {\left( {{\text{A}}_{{\text{o}}} - {\text{A}}} \right)/{\text{A}}_{{\text{o}}} } \right]$$

Beer-law Lambert states that the dye's absorbance is proportionate to its CV dye concentration.

### Ethical approval

The submitted work is original and it is not been published elsewhere in any form or language (partially or in full). All authors agreed with the content that all gave explicit consent to submit, and that they obtained consent from the responsible authorities at the institute/organization where the work has been carried out.

## Results and discussion

### X-ray diffraction (XRD)

**Figure 2:**
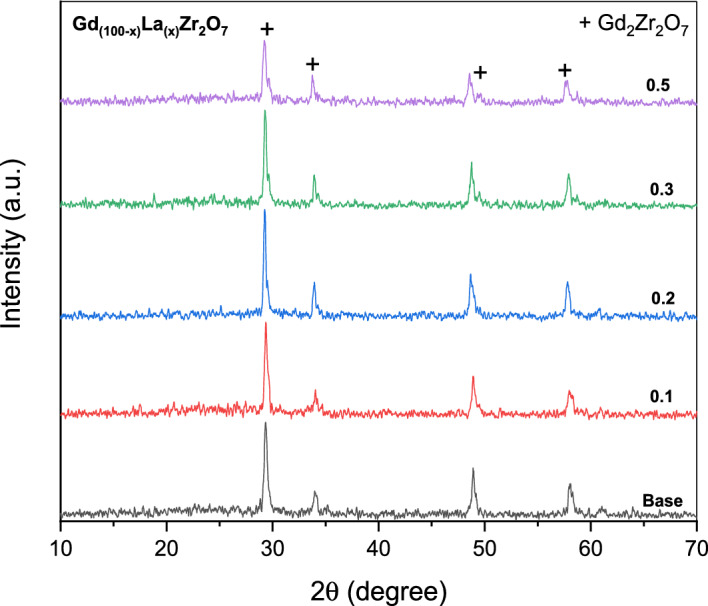
XRD of Gd_(2−x)_La_(x)_Zr_2_O_7_ nanoparticles, Gd_2_Zr_2_O_7_ (JCPD01-080–0471(c)).

X-ray diffraction pattern of Gd_(2−x)_La_(x)_Zr_2_O_7_ fabricated based on a sol–gel auto-combustion route with tartaric acid as a fuel and annealed at 1100 °C for 2 h is depicted in Fig. 2. The broad diffraction peaks associated with (111), (200), (220), (311), and (222) reflections of the defect-fluorite-structured Gd_2_Zr_2_O_7_ with space group: Fm-3 m (PDF#80–0471) at 2θ = 29.41°, 34.12°, 48.90°, 58.26°, and 61.18° were detected and these results are in a good agreement with other researches^34,35^. However, the pyrochlore superstructures at 111 (14°), 311 (28°), 331 (37°), and 511 (45°) (space group: Fd-3 m) were not found. Due to the Gd_2_Zr_2_O_7_ structure's proximity to the pyrochlore–fluorite transition's geometric border (R_Gd_/R_Zr_ = 1.46), 100% order is not recognized and is attributed to atom inversion. The movement of 10% of oxygen atoms from O1 sites to the initially unoccupied O3 sites is what causes the disordering in the oxygen sublattice.

The prepared rare earth oxide zirconate-based material is mainly. It has a pyrochlore or fluorite crystal structure. Pyrochlores generated from (La_1-x_Gd_x_)_2_Zr_2_O_7_, If the crystal structure is explained using La_2_Zr_2_O_7_ as an example, La^3+^ cation is in a specific crystallographic position, 16c (0, 0, 0) (A), Zr^4+^ The cation occupies 16d sites (1/2, 1/2, 1/2) (B sites), On the (001) plane, it alternately in the [110] direction and the [− 110] direction. An ordered lattice in which the cations of A and B are arranged in a row structure. For the oxygen ion, there are two 48f. positions.

4 $$d_{RX} = k\lambda /\beta \cos \theta$$

where the wavelength of the Cu target is 1.5406 nm, *d*_*RX*_ is the crystallite size, k = 0.9 is a correction factor to account for particle morphologies, FWHM is the full width at half maximum, and is the Bragg angle. As shown in Table 1, the average was discovered to be 27.75 nm at x = 0.1, 38.525 nm at x = 0.2, 40.76 nm at x = 0.3 and 30.85 at x = 0.5. Using Bragg's equation, *a* = *d*_*hkl*_ $\sqrt{{h}^{2}+{k}^{2}+{I}^{2}}$, the lattice parameter (a) of the synthesized Gd_(2−x)_La_(x)_Zr_2_O_7_ powders was estimated. d stands for the inter-planar spacing of the primary diffraction peak.

**Table 1:** Crystal size (nm), lattice parameter (Å), and unit cell volume (cm^3^) of Gd_(2−x)_La_(x)_Zr_2_O_7_ nanoparticles.

La^3+^ ratio, *x*	Crystal size, nm	The lattice parameter, *a,* Å	Unit cell volume, cm^3^
0.0	28.5	10.534	1.169 × 10^–21^
0.1	33.2	10.525	1.166 × 10^–21^
0.2	39.2	10.566	1.18 × 10^–21^
0.3	32.7	10.548	1.174 × 10^–21^
0.5	25.3	10.573	1.182 × 10^–21^

There are similar properties between La and Gd to be substituents for each other. The electronic structure for both of them respectively, [Xe] 5d^1^ 6s^2^ and [Xe] 4f^7^ 5d^1^ 6s^2^. Atomic radii are respectively, 195 and 233 pm. Therefore, the results of crystallite size in nm and Lattice parameters for Gd_(2−x)_La_(x)_Zr_2_O_7_ are very reasonable.

### Fourier transform infrared (FTIR)

**Figure 3:**
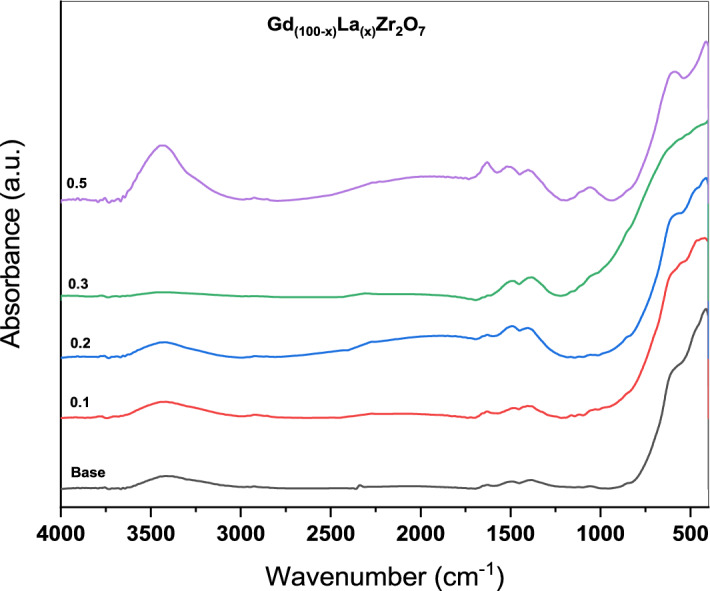
FTIR of Gd_(2−x)_La_(x)_Zr_2_O_7_ nanoparticles.

The synthesized sample's FT-IR spectra are shown in Fig. 3. It should be observed that the absorption peak between wave numbers 1640 cm^−1^ and 3425 cm^−1^ is connected to the bending and vibrating of OH in water attributable to the water that has been absorbed from the atmosphere^34^. The absorption bands at 547 and 1425 cm^−1^ are caused by Gd–O vibrations. The recognizable peak at 715 cm^−1^ correlates to the Zr–O–Zr stretch-related vibrations. The stretching vibration of the M–O bond (M=Zr–Gd) produces a distinctive peak at 847 and 666 cm^−1^. Peaks at 1505, 1398, and 1080 cm^−1^ are associated with Zr–OH vibrations^35^.

### TEM

**Figure 4:**
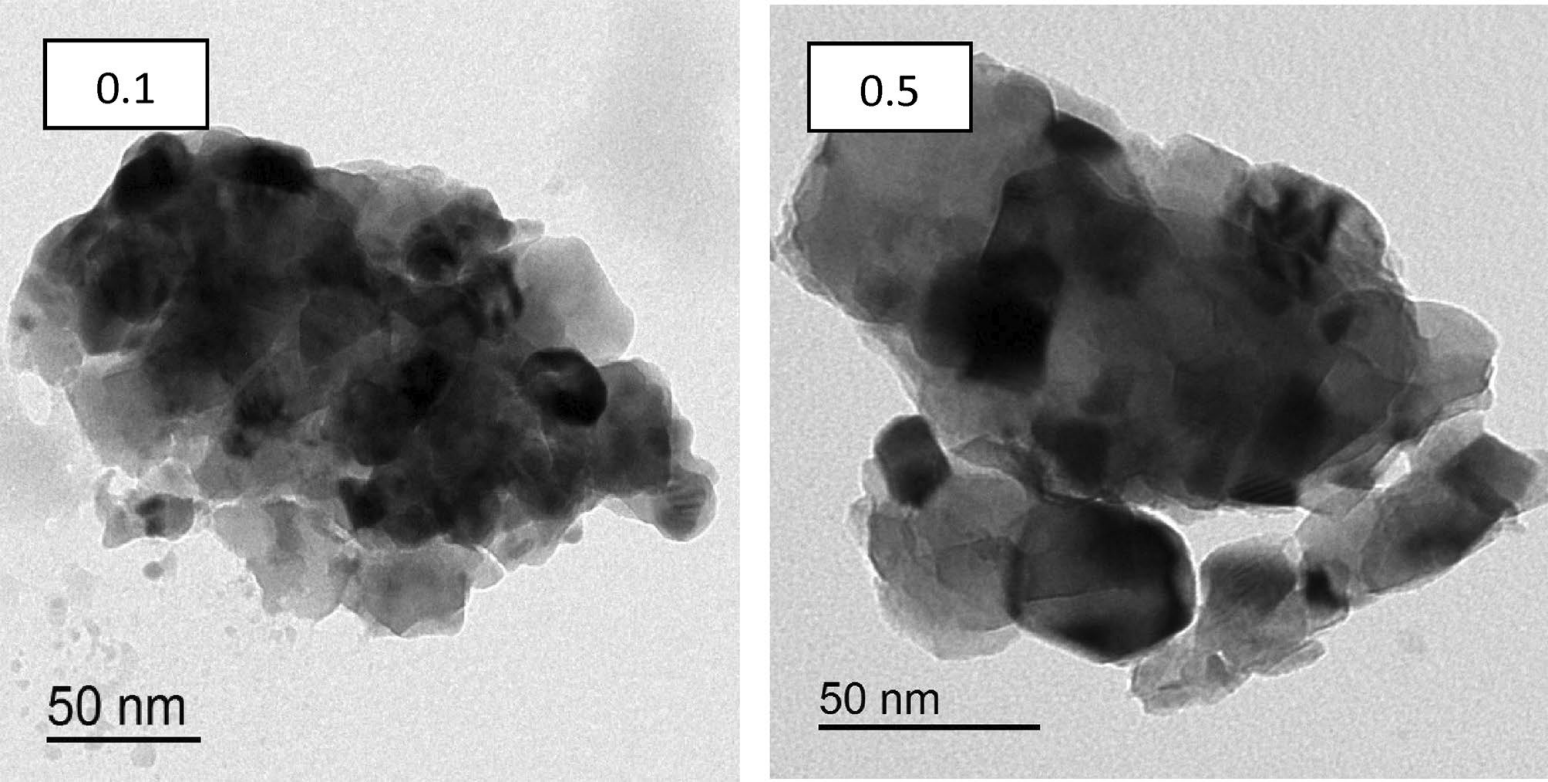
TEM micrographs of Gd_(2−x)_La_(x)_Zr_2_O_7_ nanoparticles.

The fracture surface of Gd_(2−x)_La_(x)_Zr_2_O_7_ where x = 0.1 and 0.5, is distinguished using TEM images presented in Fig. 4. The grain growth rate increased with evaluated La^3+^ ion concentration. The grain displays a cubic-like structure with a degree of agglomeration. The particle size was increased from 20 to 50 nm with a further increase in La^3+^ ions. Such results can be explained on the basis of the change of ionic radius between La^3+^ and Gd^3+^ ions. The SAED diffraction patterns indicated in Fig. 4 were in agreement with a defect-fluorite structured fluorite structure.

**Figure 5:**
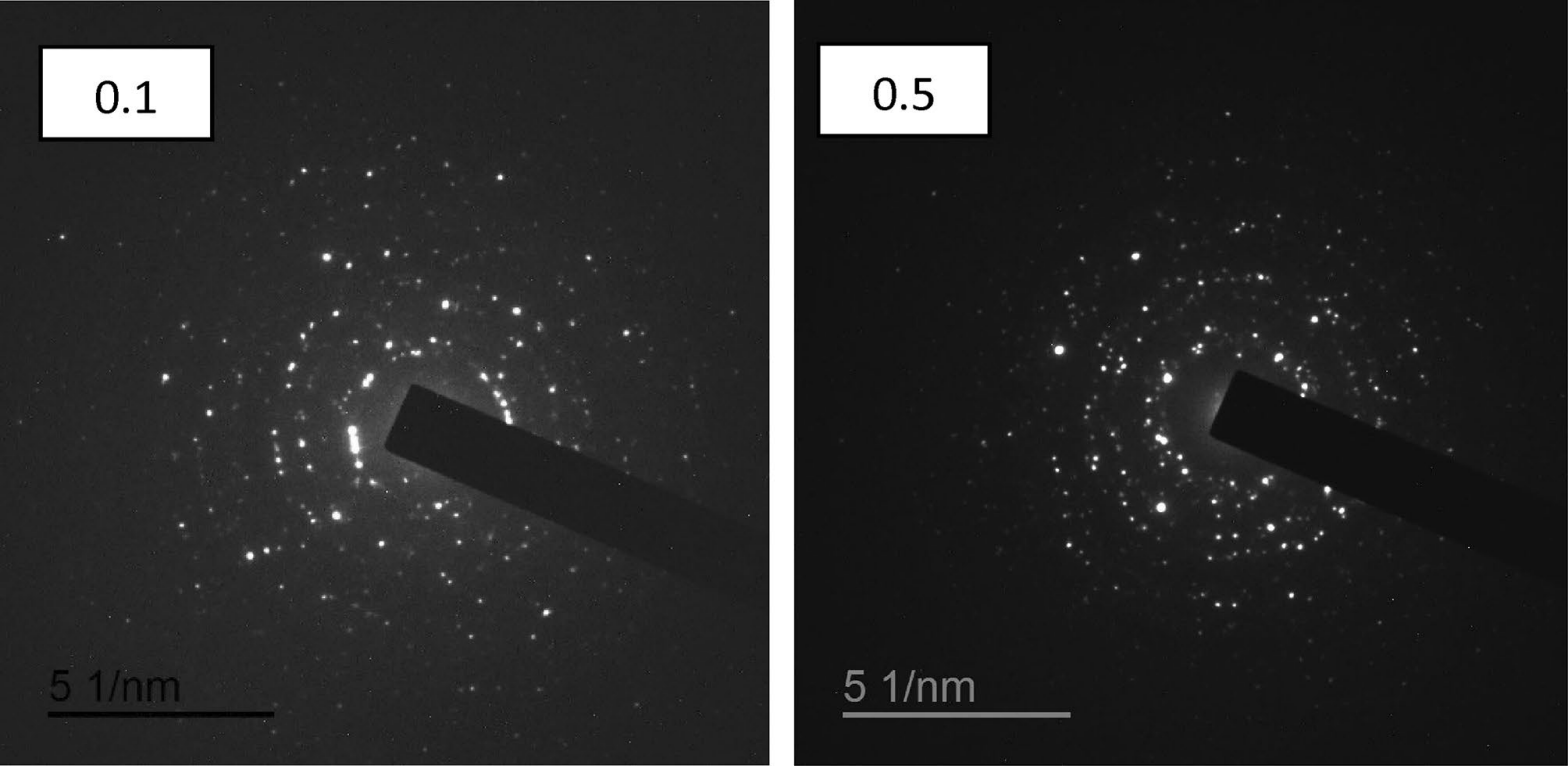
Electron Diffraction pattern of Gd_(2−x)_La_(x)_Zr_2_O_7_ nanoparticles.

Figure 5 indicates the Electron Diffraction pattern of Gd_(2−x)_La_(x)_Zr_2_O_7_ nanoparticles which proves the results of the X-ray and both prove the crystalline features of the prepared samples.

### Optical properties

**Figure 6:**
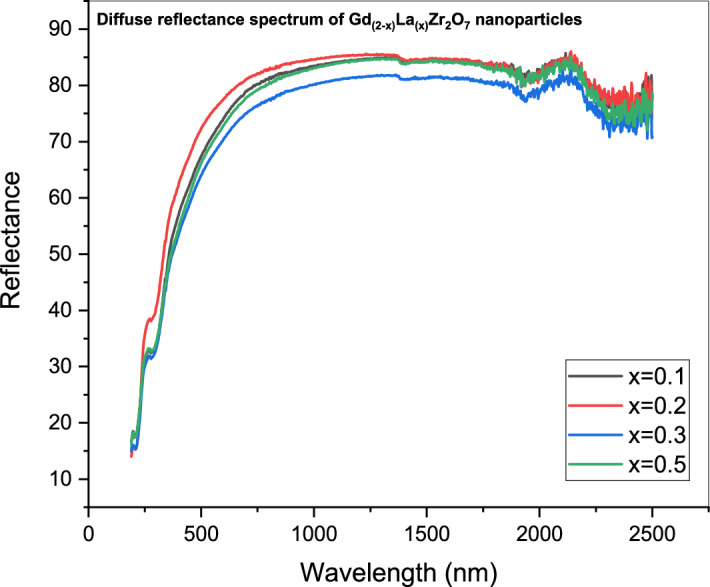
Diffuse reflectance spectrum of Gd_(2−x)_La_(x)_Zr_2_O_7_ nanoparticles.

Gd_(2−x)_La_(x)_Zr_2_O_7_ phosphors' optical band gap is identical to that of Gd_(2−x)_La_(x)_Zr_2_O_7_ itself based on the Kubelka–Munk theory and diffuse UV–Vis spectral reflectance Fig. 6 illustrates the sample's diffuse reflectance spectrum.

**Figure 7:**
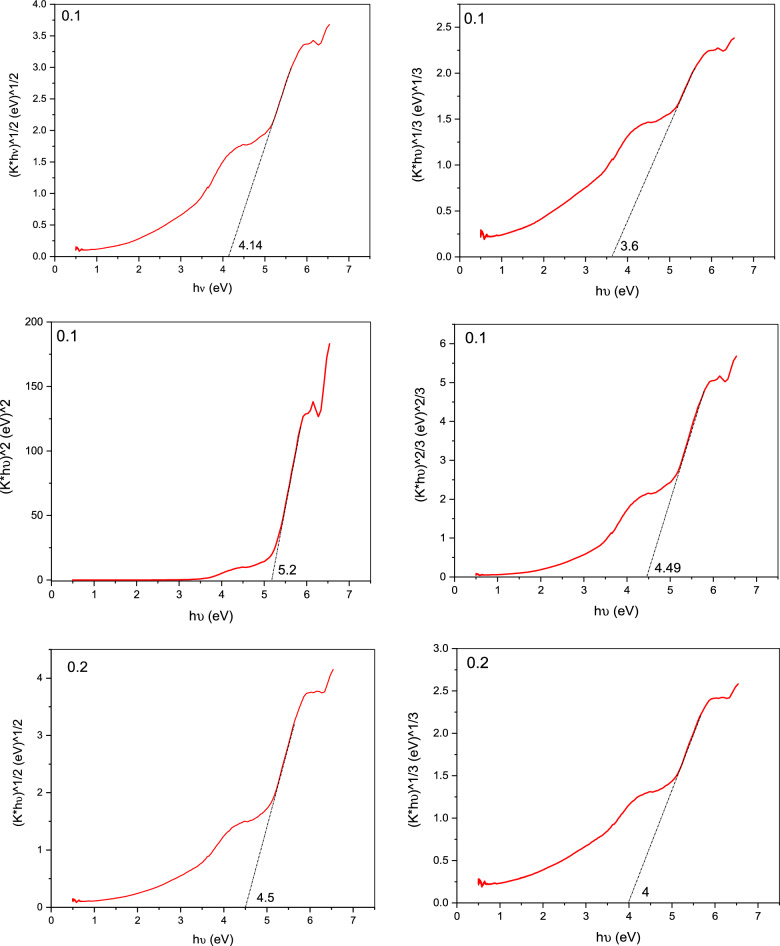

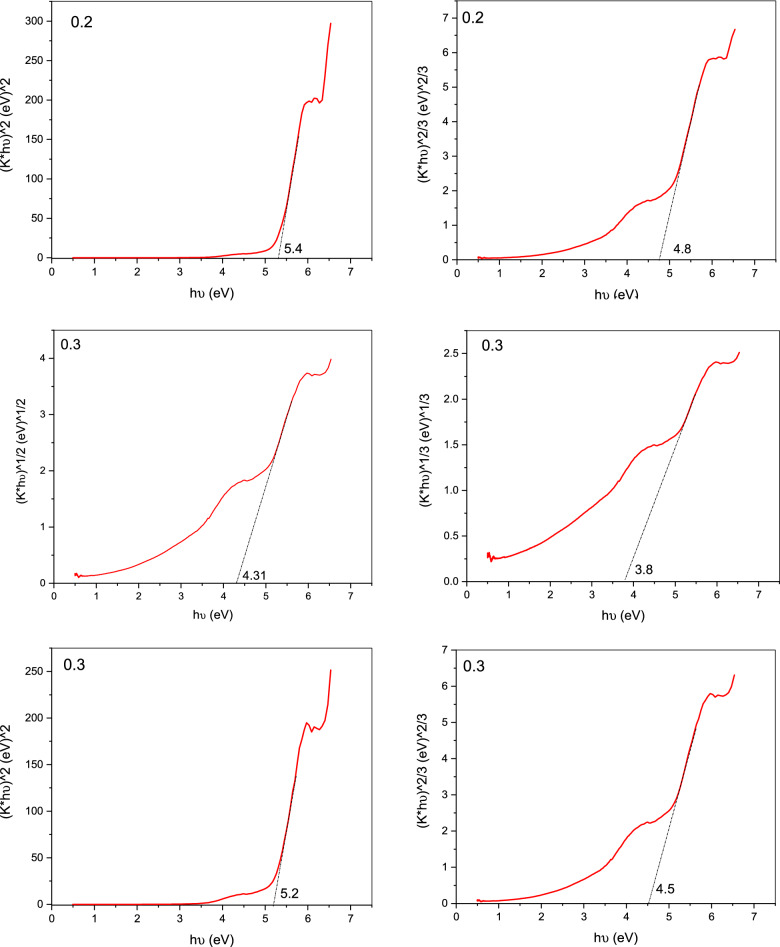

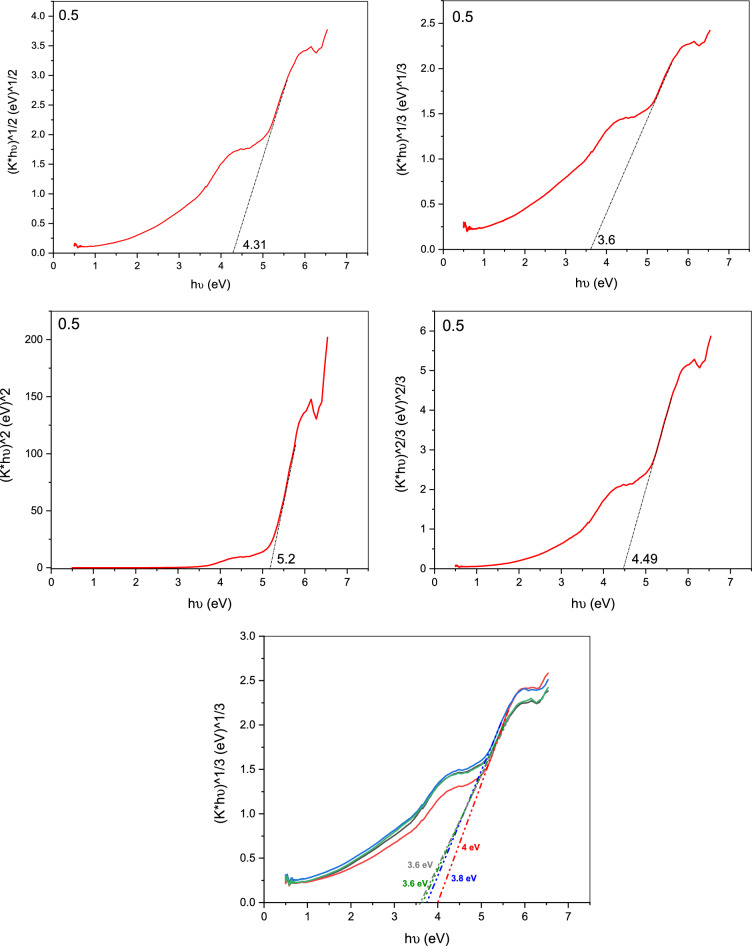
Kubelka–Munk plot of pure Gd_(2−x)_La_(x)_Zr_2_O_7_.

From the measurement of UV/Vis. analysis, it is noticed that an increase in the absorbance spectra of the contaminated water (crystal violet solutions) with the increase in purification time. The Kubelka–Munk (K–M) function is used to transform reflectance spectra to comparable absorption spectra as shown in Fig. 7.

The descriptions of the following equations, which were obtained from the literature for the Eg calculation, indicate the sort of transition that was taken into consideration.

$$\alpha \left( {{\text{h}}\upsilon } \right) \approx {\text{B}}\left( {{\text{h}}\upsilon - {\text{E}}_{{\text{g}}} } \right)^{{\text{n}}}$$

n = 2 for an indirect permitted transition (plotted as h)^1/2^ vs. E), n = 3 for an indirect prohibited transition (plotted as h)^1/3^ versus E), n = 3/2 for a direct forbidden transition, and n = 1/2 for a direct allowed transition. (Eg signifies the band gap (eV), h the Planck's constant (J.s), B the absorption constant, v the light frequency (s^−1^), and (α) the extinction coefficient, which is proportional to F. (R). The best linear fit in the absorption spectra can be used to experimentally calculate the n value for a particular transition using several formulas.

Below the conduction band, which reached up to the valence band maximum, the Gd d states were seen. La_2_Zr_2_O_7_'s La d states are connected to Gd d states that extend in the direction of the band gap. In the Gd_16_Zr_16_O_56_ system, the valence band is mostly contributed by O p states, whereas the conduction band is produced by Zr d states. The Gd d states have a partial coupling to the valence and conduction bands and a partial band gap appearance. Thermal conductivity variations of La_2_Zr_2_O_7_ and Gd_2_Zr_2_O_7_ with varying La/Gd concentrations^36^.

### Visible spectrophotometer

**Figure 8:**
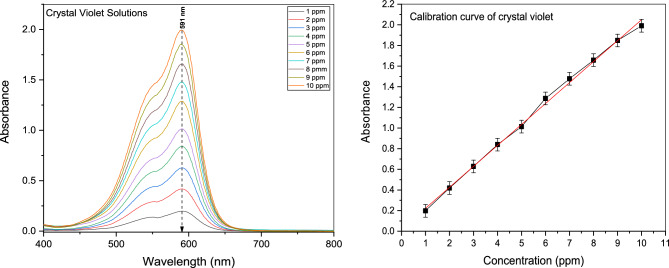
Calibration curve of crystal violet solutions.

Figure 8 illustrates the final calibration curve used to calculate the concentration of crystal violet solutions during the purification process using the information on the solutions' wavelength-dependent absorbance (591 nm).

The concluded equation is:

5 $${\text{Conc}}{.} = \frac{{{\text{Absorbance}} + 0.0202}}{0.20289}\quad {\text{For}}\;{\text{Crystal}}\;{\text{Violet}}$$

**Figure 9:**
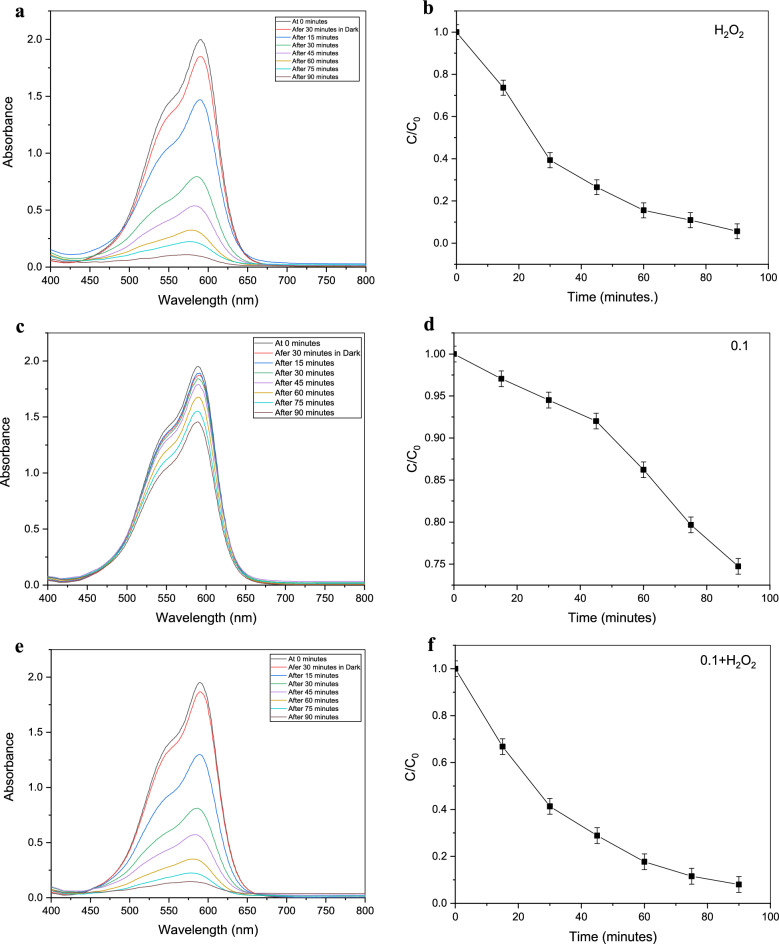

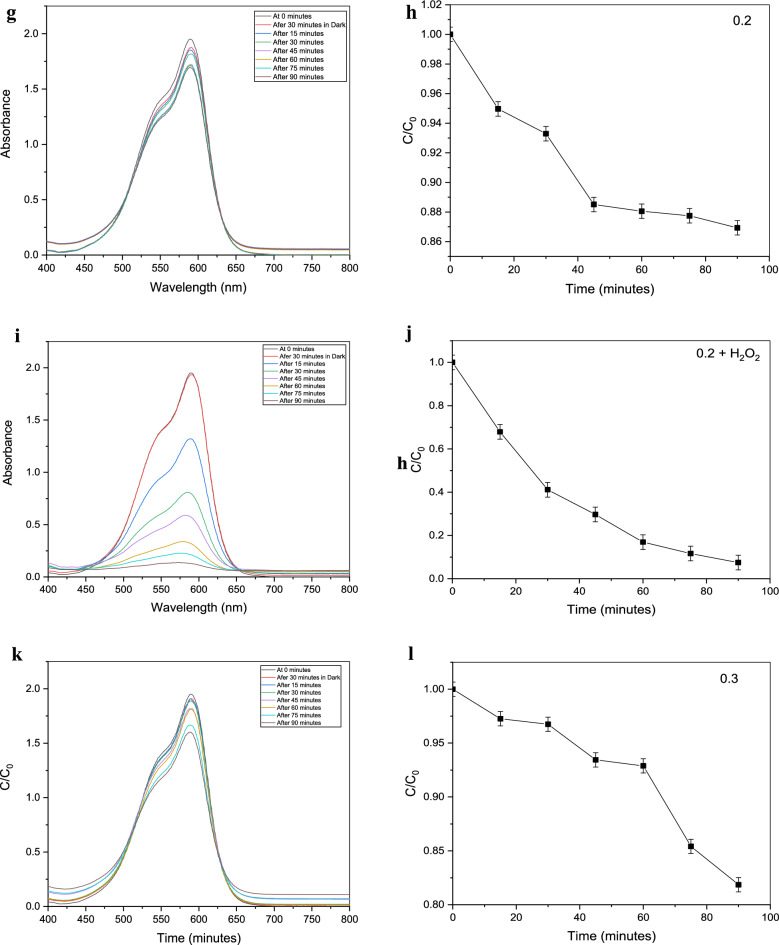

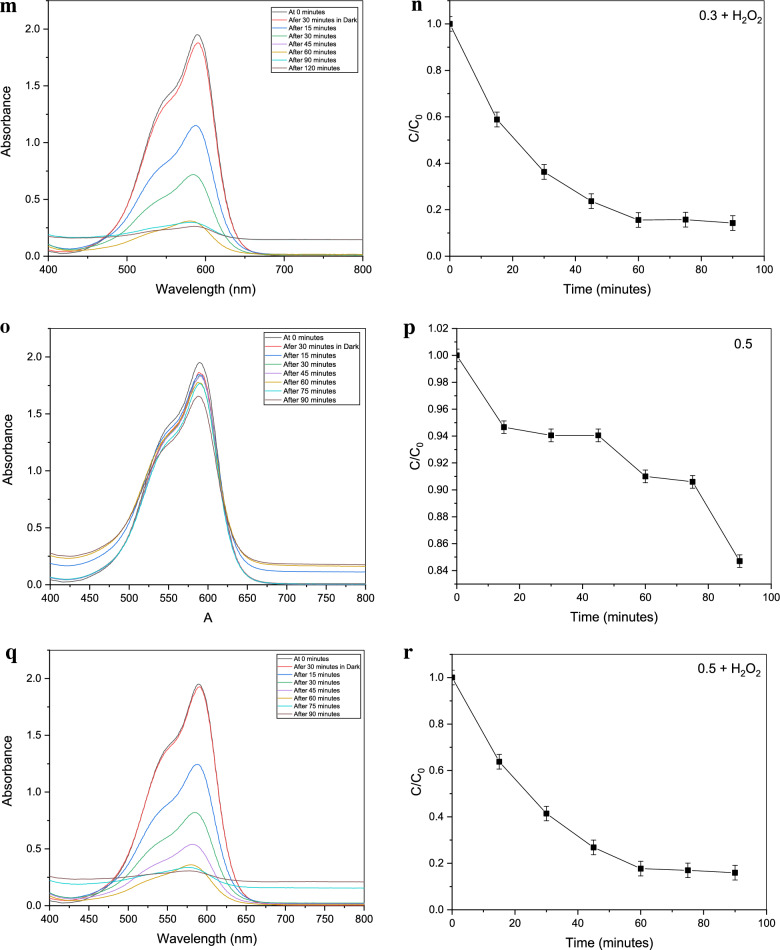

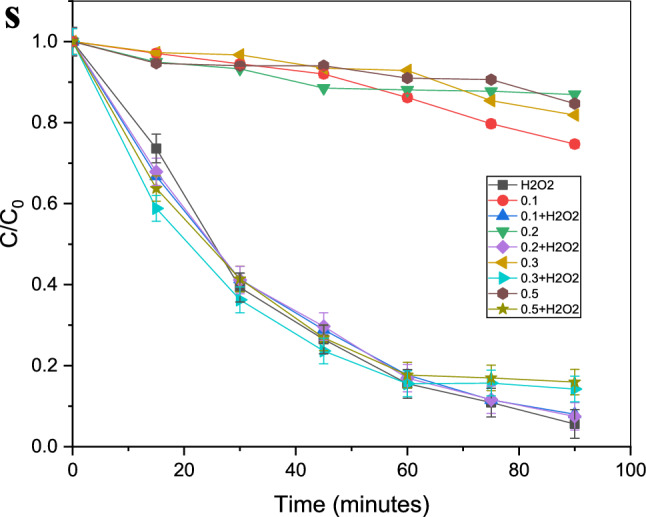
Absorbance of 10 ppm solution of crystal violet after treatment with (**a**)H_2_O_2_ only, 0.05 gm of Gd_(2−x)_La_(x)_Zr_2_O_7_ without H_2_O_2_ where (**c**) x = 0.1, (**g**) x = 0.2, (**k**) x = 0.3, (**o**) = 0.5, Also with H_2_O_2_ where (**e**) = 0.1, (i) = 0.2, (**m**) = 0.3, (**q**) = 0.5 and (**b**–**r**) are concentrations conversion C/C_0_ versus time for degradation process where (**s**) is the figure of combined data.

According to the examination of the visible spectrophotometer measurements, the absorbance spectra of the contaminated water (crystal violet solutions) increase as the purification time increases. From the Crystal Violet solution, known concentrations of 1, 2, 3,…, and 10 ppm were generated. By using a visible spectrophotometer to measure their absorbance spectrum, a calibration curve for those concentrations was created, and an equation was then developed to use their absorbance to compute the unknown amounts that would follow from the purification procedure, as shown in Fig. 9.

It was put through a photocatalyst test using crystal violet solution (10 ppm) with intervals of 15 min between 0 and 90 min. Gd_(2−x)_La_(x)_Zr_2_O_7_ nanoparticles were utilized as a catalyst for the Fenton-like reaction to purify by being exposed to hydrogen peroxide solution (32 watts). Different researches have been carried out with lots of materials for decolorization of crystal violet by photodegradation or even Fenton-like reaction as shown in Table 3 and Gd_(2−x)_La_(x)_Zr_2_O_7_ nanoparticles proved a great efficiency compared with these materials.

It is worth noting that Gd_(2−x)_La_(x)_Zr_2_O_7_ has never been used with the same composition in the photocatalysis process, according to our consideration. According to the literature survey, there are two types of researches or more that prepared the same elements used in our research, but with a different composition, the basis of which is La_2_Zr_2_O_7_, with substitutions of gadolinium, unlike our research in which G_2_Zr_2_O_7_ is the base. In addition, this composition was not used in the process of water purification, and this indicates that our research is one of the first research that used Gd_(2−x)_La_(x)_Zr_2_O_7_ with the same method in the photocatalysis process^37,38^.

The purification procedure was performed under identical conditions using gadolinium zirconium oxide alone, hydrogen peroxide alone again, and then both of them combined with 1 ml of hydrogen peroxide and 0.025 g of pyrochlore in 50 ml of crystal violet solution. It was observed that pyrochlore alone demonstrated a respectable efficiency and that this efficiency was increased by combining it with hydrogen peroxide. They showed rapid degradation of crystal violet, going from a concentration of 10 ppm to 0 ppm in no more than 90 min. By comparing with other researches, most of them used the same percent of photocatalyst in wastewater treatment process^39^.

Table 2 shows the values of change in concentration or relative concentration (C/C_0_) for crystal violet solutions (50 ml) during the purification process. The photocatalysis process has been carried out in three parallel categories. The first of them included the photocatalysis of crystal violet solution (50 ml) by using 1 ml of hydrogen peroxide alone (H_2_O_2_). The second has proceeded with Gd_(2−x)_La_(x)_Zr_2_O_7_ pyrochlore in addition to hydrogen peroxide. The last one was carried out by using Gd_(2−x)_La_(x)_Zr_2_O_7_ pyrochlore alone (Gd_(2−x)_La_(x)_Zr_2_O_7_ nanopowder). Moreover, all of these categories have proceeded under the influence of ultraviolet radiation in which the sample was taken for analysis every 15 min.

**Table 2:** Changes in concentration values or relative concentrations (C/C_0_) for crystal violet solution (contaminated water) during photocatalysis process under the irradiation of UV by using 1- Hydrogen peroxide (H_2_O_2_) alone; 2- Gd_(2−x)_La_(x)_Zr_2_O_7_ catalyst nanopowder alone; Where X = 0.1, 0.2, 0.3, and 0.5; 3- H_2_O_2_ plus Gd_(2−x)_La_(x)_Zr_2_O_7_ nanopowder.

C/C_0_ values of crystal violet solution during photocatalysis with									
Degradation Time (minutes)	H_2_O_2_ alone	Gd_(1.9)_La_(0.1)_Zr_2_O_7_ alone (X = 0.1)	Gd_(1.9)_La_(0.1)_Zr_2_O_7_ plus H_2_O_2_	Gd_(1.8)_La_(0.2)_Zr_2_O_7_ alone (X = 0.2)	Gd_(1.8)_La_(0.2)_Zr_2_O_7_ plus H_2_O_2_	Gd_(1.7)_La_(0.3)_Zr_2_O_7_ alone (X = 0.3)	Gd_(1.7)_La_(0.3)_Zr_2_O_7_ plus H_2_O_2_	Gd_(1.5)_La_(0.5)_Zr_2_O_7_ alone (X = 0.5)	Gd_(1.5)_La_(0.5)_Zr_2_O_7_ plus H_2_O_2_
0	1	1	1	1	1	1	1	1	1
15	0.73617	0.971	0.66805	0.94966	0.67872	0.97254	0.58824	0.94661	0.63755
30	0.39313	0.945	0.41337	0.93289	0.41134	0.96746	0.36254	0.94051	0.41439
45	0.26493	0.92	0.28883	0.88511	0.29697	0.93441	0.23648	0.94051	0.2685
60	0.15553	0.862	0.177	0.88053	0.16938	0.92882	0.15565	0.91001	0.177
75	0.109	0.797	0.11549	0.87748	0.11651	0.8541	0.15718	0.90595	0.16988
90	0.05603	0.747	0.08042	0.86935	0.07483	0.81851	0.14243	0.84698	0.15972

Photocatalysis efficiency is a vital parameter that should be taken into consideration. In the case of photocatalysis with Gd_(2−x)_La_(x)_Zr_2_O_7_ nanoparticles alone, it proved an average efficiency according to values of relative concentration (C/C_0_) in Table 2.

For the catalysis with pyrochlore in addition to hydrogen peroxide, the interpretation is slightly different, as it is done with a process called Fenton-Like Reaction, and in this case, hydrogen peroxide is indispensable. Similar to the situation of purification with hydrogen peroxide alone, this case is characterized by its high efficacy and a rate of removal that approaches zero in a period time that is likewise close to 90 min. Although it may appear superficially that Gd_(2−x)_La_(x)_Zr_2_O_7_ pyrochlore has no function as long as we get the same percentage with or without it, the reality is rather different. The efficiency of the purification process in the case of pyrochlore with hydrogen peroxide is better than hydrogen peroxide alone, even if we get the same efficiency approximately at the end of the 90 min purification period according to the values of the relative concentrations^5^. Thus, It was found that the efficiency of the Fenton-Like approach, which comprises pyrochlore and hydrogen peroxide, is greater than pyrochlore alone at the start of the process. Furthermore, we employed a very low concentration of pyrochlore, up to 0.025 g per 50 ml of pollutant. If this percentage is increased, the purification process will be accelerated. and thus proves the efficiency of pyrochlore in the degradation process by employing a Fenton-like reaction (Table 3).

**Table 3:** Efficiency of different materials against crystal violet dye solution by Photodegradation and Fenton-like reactions.

Catalyst	Type of reaction	Results	References
10%NiO–ZnO	Photodegradation	100% removal after 180 min	Saeed et al.^42^
rGO/Fe_3_O_4_/CdS	Photodegradation	95.4% efficiency	Alwan et al.^43^
Octadecylamine capped cadmium nanoparticles CdS	Photodegradation	75–84% efficiency	Oladipo et al.^28^
ZnO/CAN	Photodegradation	92% efficiency	Messaoudi et al.^29^
PM-Fe/Ni/H_2_O_2_	Fenton like reaction	91.86% efficiency	Liu et al.^27^

In this context, the photocatalytic method is characterized by the separation of electron/hole pairs (e^−^/h^+^) and the separation of photogenerated carriers can be connected to the crystalline phase, the aspect ratio of nanopowders, electronic structure of the facets, and defects. The reaction Eqs. (6)–(19) can be used to represent the mechanism of photocatalytic degradation of crystal violet dye^40^.

The photogenerated VB (h^+^) nanoparticles combine with either H_2_O or OH^-^ to form OH^·^ via the following reaction with assessed activity and indirectly decay the dye molecule^41^.

6 $${\text{Gd}}_{{({2} - {\text{x}})}} {\text{La}}_{{({\text{x}})}} {\text{Zr}}_{{2}} {\text{O}}_{{{7} }} + h\upsilon \to {\text{h}}_{{{\text{VB}}}}^{ + } + {\text{e}}_{{{\text{CB}}}}^{ - }$$

7 $${\text{h}}^{ + } + {\text{e}}^{ - } \to {\text{heat}}$$

8 $${\text{H}}_{{2}} {\text{O}} + {\text{h}}_{{{\text{VB}}}}^{ + + } \to {\text{H}}^{ + } + {\text{OH}}^{ - }$$

9 $${\text{h}}_{{{\text{VB}}}}^{ + } + {\text{OH}}^{ - } \to {\text{OH}}$$

As a result of the presence of hydroxyl groups, water, and oxygen at the surface of (Gd_(2−x)_La_(x)_Zr_2_O_7_ nanoparticles, the electrons (e^−^) in the conduction band react with species absorbed on the (Gd_(2−x)_La_(x)_Zr_2_O_7_ surface, free radicals to generate O_2_^·−^, and following the subsequent steps, it results in the generation of OH^·^ radicals.

10 $${\text{e}}_{{{\text{CB}}}}^{ - } + {\text{O}}_{{2}} \to {\text{O}}_{{2}}^{ - }$$

11 $${\text{O}}_{{2}}^{ - } + {\text{HO}}_{{2}} + {\text{H}}^{ + } \to {\text{H}}_{{2}} {\text{O}}_{{2}} + {\text{O}}_{{2}}$$

12 $${\text{H}}^{ + } + {\text{O}}_{{2}}^{\cdot - } + {\text{eCB}}^{ - } \to {\text{HO}}^{\cdot}$$

13 $${\text{O}}^{\cdot - } + {\text{OH}}_{{2}}^{\cdot} \to {\text{OHO}}_{{2}}^{\cdot} + {\text{O}}^{ - }$$

14 $${\text{HO}}_{{2}}^{\cdot} \to {\text{H}}_{{2}} {\text{O}}_{{2}} + {\text{ O}}_{{2}}$$

15 $${\text{H}}_{{2}} {\text{O}}_{{2}} + {\text{e}}_{{{\text{CB}}}}^{ - } \to {\text{OH}}^{\cdot} + {\text{OH}}^{ - }$$

16 $${\text{H}}_{{2}} {\text{O}}_{{2}} + \to {\text{2OH}}^{\cdot}$$

Finally, the produced agents degraded the dye pollution.

17 $${\text{CV}} + {\text{O}}_{{2}}^{\cdot - } \to {\text{CV}}^{\cdot - } \to {\text{product}}$$

18 $${\text{CV}} + {\text{OH}}^{\cdot} \to {\text{CV}}^{\cdot} \to {\text{product}}$$

19 $${\text{CV}} + {\text{OHO}}_{{2}}^{\cdot} \to {\text{CV}}^{\cdot} \to {\text{product}}$$

It is known that the use of H_2_O_2_ in combination with the photocatalyst was shown to be effective in eliminating hazardous organic species from water. H_2_O_2_ can hinder the surface recombination of electron–hole pairs and stimulate the production of hydroxyl radicals^5^.

Due to its reaction with the valence band holes and conduction band electrons in photocatalytic systems, H_2_O_2_ raises the concentration of the ^·^OH and ^·^O_2_ radicals. H_2_O_2_ can react with ^·^OH, HO_2_^·^, or ^·^O_2_ radicals to produce further radicals (Eqs. 20–25). The oxidative species interact with one another or with the holes they create to produce O_2_ (Eqs. 26, 27). Additionally, UV light exposure to H_2_O_2_ results in the formation of hydroxyl radicals (Eq. 28).

20 $${\text{H}}_{{2}} {\text{O}}_{{2}} + {\text{ e}}^{ - }_{{{\text{CB}}}} + {\text{ H}}^{ + } \to^{ \cdot } {\text{OH}} + {\text{H}}_{{2}} {\text{O}}$$

21 $${\text{H}}_{{2}} {\text{O}}_{{2}} + {\text{e}}^{ - }_{{{\text{CB}}}} \to^{ \cdot } {\text{OH}} + {\text{OH}}^{ - }$$

22 $${\text{H}}_{{2}} {\text{O}}_{{2}} + {\text{h}}^{ + }_{{{\text{VB}}}} \to {\text{HO}}_{{2}} + {\text{H}}$$

23 $${\text{H}}_{{2}} {\text{O}}_{{2}} + {\text{HO}}_{{2}}^{ \cdot } \to^{ \cdot } {\text{OH}} + {\text{H}}_{{2}} {\text{O}} + {\text{O}}_{{2}}$$

24 $${\text{H}}_{{2}} {\text{O}}_{{2}} +^{ \cdot } {\text{O}}_{{2}}^{ - } \to^{ \cdot } {\text{OH}} + {\text{OH}}^{ - } + {\text{O}}_{{2}}$$

25 $${\text{H}}_{{2}} {\text{O}}_{{2}} +^{ \cdot } {\text{OH}} \to {\text{H}}_{{2}} {\text{O}} + {\text{HO}}_{{2}}^{ \cdot }$$

26 $${\text{HO}}_{{2}}^{ \cdot } +^{ \cdot } {\text{OH}} \to {\text{H}}_{{2}} {\text{O}} + {\text{O}}_{{2}}$$

27 $${\text{HO}}_{{2}}^{ \cdot } + {\text{h}}^{ + }_{{{\text{VB}}}} \to {\text{O}}_{{2}} + {\text{H}}^{ + }$$

28 $${\text{H}}_{{2}} {\text{O}}_{{2}} + {\text{h}}\nu \to {2}^{ \cdot } {\text{OH}}$$

The great differences are mainly derived from photo-induced thermal energy, which in-situ heating active sites to lower the H_2_O_2_ activation barrier^44^.

## Conclusion

In summary, Gd_(2−x)_La_(x)_Zr_2_O_7_ with a variable Gd/La ratio was successfully prepared using a sol–gel auto-combustion approach based on tartaric acid as fuel. The La-substituted Gd_2_Zr_2_O_7_ nanopowders existed in a defective fluorite phase. The crystallite size of the synthesized nanoparticles was found to be from 28.5 to 39.2 nm. Unit cell volume was found to be 1.2 × 10^–21^ cm^3^. The grain size of Gd_(2−x)_La_(x)_Zr_2_O_7_ increased with increasing La^3+^ ion content. Most of the synthesized particles demonstrate a cubic-like structure. The diffuse reflectance spectrum confirmed the synthesized particles are highly transmissive. The band gap energy of the synthesized Gd_(2−x)_La_(x)_Zr_2_O_7_ samples was found to be (4–3.6 eV). Finally, the synergistic effect of Gd_(2−x)_La_(x)_Zr_2_O_7_ and H_2_O_2_ possessed an advanced oxidation reaction with high efficiency towards organic pollutants degradation, such as crystal violet dye, in a cost-effective way and a short time.

## Data Availability

All data generated or analyzed during this study are included in this article.
